# Treatment of Cervical Borders

**Published:** 1898-09

**Authors:** Safford G. Perry

**Affiliations:** New York


### TREATMENT OF CERVICAL BORDERS.[1]

BY SAFFORD G. PERRY, D.D.S., NEW YORK.

From whatever point it may be considered, the cervical border presents the weakest portion of every approximal cavity. It is inaccessible, it is anatomically weak, and, above all in importance, it is, from its position, most vulnerable to the insidious agents that destroy the teeth.

---

[1] Read before the Academy of Stomatology, April 26, 1898.

In considering this border, then, we have to contend with the most difficult conditions ever met with in the care of the teeth, for it goes without saying that the proximal cavities, as a class, are the most troublesome in the mouth. We can never be old enough, wise enough, patient enough, and accurate enough to care for this border too perfectly.

There is such great variety in the conditions found here that the operator is constantly meeting with cases that enlist his keenest interest and stimulate his highest ambition.

Logically, the first thing to consider is the preparation of this border for the reception of whatever filling-material may be used. Here we meet at once with an anatomical condition that must receive most careful attention.

I refer, of course, to the increasing thinness of the enamel as the cavity extends towards the root of the tooth. Of course, we must cut until we have reached solid structure, no matter how far we have to go, but here we are met by the question, What is solid structure? Strictly speaking we might cut up on the root, even above the border of the enamel, and not find it.

It may be answered that we had better do so if we cannot. To this I assent in most cases, but not by any means in all, for some teeth are in a condition that a solid border could not be found without going far under the gum, and then the cavity could not be filled and finished in such a manner that the gum would ever again remain perfect along this border.

I am convinced that ruthless disturbance of the gum is given little heed to by most operators,—by many, who still with a separating file destroy the perfect contour, in spite of all that has been written and said against this practice during the last twenty-five years, and by others who are such radical contourists that they cannot rest until they have cut even small cavities up under the gum, and made large fillings that shall have free edges, and that, along the cervical border, almost invariably leave a slightly disturbed condition of the gum, which bleeds readily when the floss silk or toothpick, and sometimes even the brush, are used.

More than twenty years ago, in an article on the treatment of the proximate surfaces, I advocated, in some cases, this free cutting of even small cavities, but I never supposed the system would be applied generally and to teeth of good structure. I have repeatedly seen cases from the hands of extreme contourists, where every filling on the approximate surfaces of bicuspids and molars ran far under the gum, and where that tissue at the cervical border was ready to bleed freely at the slightest touch. I will grant that such fillings are safe,—none could be safer,—but in many of the cases I have seen the teeth of such good quality that I did not consider such radical operations necessary, for the teeth would have been safe with the filling that did not reach under the gum, and there would have been no disturbance of that tissue, and, besides all this, there would have been less display of gold, and infinitely less work and pain in performing the operations. In cutting, therefore, along the cervical border I believe it to be good practice to avoid going under the gums if the teeth are of good quality, and the general conditions in the mouth are not destructive. Please notice that I say in teeth of good structure. Yet, lest I be caught tripping here, in view of Dr. Black's experiments, which would seem to indicate that all teeth are nearly of the same structure, let me say, in destructive mouths.

Then, in cutting, the diminishing enamel must be considered, and in cutting a little more in the hope of getting a better border, there is danger of reaching such thin enamel, that it may be a question if there has been any gain by this more thorough cutting. It seems to me there is no ordinary operation where nicer care and judgment are called for than in the preparation of this cervical border. It is literally a "ragged edge," which one may approach too timidly, or over which one may too boldly go.

In the preparation of it for gold, there must be more thorough cutting than for any of the plastics, because the force required in condensing the gold might check and disturb an enamel border, which might be firm enough to receive a plastic. Then, too, the preparation must depend somewhat upon the gold to be used.

If cohesive gold is to be used there must be made one or two retaining points, or a retaining groove must be cut across the whole cervical border. If soft gold is selected, it may be that no retaining point or undercut will be needed.

It is probably safe to say that no dentist of experience can be found who would not be glad if he could be free from the necessity of ever making a retaining pit, or cutting a retaining groove in any tooth to be filled with gold. Then would be saved the devitalization of the border of the cavity caused by cutting the fibrillæ, the filling of a pit or groove which is likely to be imperfectly done, and the greater danger of crushing an enamel edge which has been weakened by the making of this pit or groove. But beggars cannot be choosers, and there must be retaining grooves in every cavity to be filled with gold.

Luckily, they need not always be at the cervical border, since the introduction of the matrix, which, making the fourth wall, becomes a great help in holding the gold in place, so that the retaining pit or the groove can be dispensed with in starting the filling, and when this is the case the mallet can be used without the fear of crushing the enamel edge, and with the certainty of making a perfect adaptation of the gold.

But if the pit or groove is omitted, there must be great watchfulness lest the gold may move after it has been condensed along the cervical border. I have had failures at this border years after when I felt certain that the gold had moved slightly without my notice before I had it securely keyed in by packing against the side walls of the cavity.

In a cavity which has been prepared along the cervical border without a pit or retaining groove, a mat of soft gold can be laid, extending a little beyond the cavity, and a matrix applied, pushing the protruding gold towards the neck of the tooth, and having the effect of holding the mat in place, and making it possible to get perhaps a more perfect adaptation of the gold to the extreme enamel edge of the tooth. My attention was first called to this by Dr. J. A. Woodward, and it has proved to be a point of practical value.

If one can have both hands free, so that the gold can be held rigidly in place for the upper third of the cavity, or until there is no chance of its moving from its place, the absence of retaining pits or grooves along the cervical border may be a clear gain. This may involve the need of an attendant to hold the rubber out of the way and reflect the light, and perhaps use the mallet. If the operator prefers to use the mirror in packing the gold, or to reflect the light and select a matrix that can be fixed to the tooth, a pit or groove at the cervical border may be necessary, since the left hand is not free to hold the first pieces of gold in place.

This feeling of certainty that the gold will stay exactly where it is placed is of great importance, and should make one pause in the preparation of a cavity to make mental comparison of the two systems. My own belief is that an operator should be as ready with one method as with the other.

In what I have said thus far, I have in mind the use of a soft gold such as Abbey's, Morgan and Hasting's, or Rowan's soft cylinders; either of these by slight annealing becomes cohesive enough to be packed as cohesive gold, and yet without entirely losing their soft quality.

A gold of this kind answers the purpose so perfectly that I

never use a strictly cohesive gold along this border. The introduction of the de Trey, as well as the Steurer gold, which seems to meet with much favor in some quarters, lessens the need of retaining pits, as this form of gold stays where it is placed better than any other, and can be used with less care in holding the first few pieces in place. Examination of the surfaces of fillings made of these golds shows an accurate adaptation, and it may be that they may yet prove to be most suitable along the cervical border. Any method that will encourage or allow delicacy in the treatment of this border is to be hailed as an advance. We appreciate this when we think of the days of cohesive gold and the sledge-hammer blows of the mallet.

It is really curious to notice how long a time is required for our profession to become conscious of the fact that the walls of even good teeth are frail and very destructible, and that cohesive gold is a very hard and unmanageable substance to use against them. The power of instinctive discrimination is not given to all, and perhaps it is not strange that we see so much poor work.

In the beginning I spoke of the inaccessibility and vulnerability of the cervical border. There is a qualifying condition which I have never heard mentioned. In every approximal cavity, in beginning the filling, the end of the plugger forces the gold squarely against the cervical border, and as the instrument is applied at right angles, the gold can be most easily and accurately adapted to the tooth with least force.

As the curve of the cavity is reached on either the lingual or the buccal aspect, this directness of application of force is lost, and this doubtless accounts for the fact that so many failures commence at this point on either the buccal or lingual sides of the tooth. By the time the cavity is about one-third filled and until finished the instrument is packing along the sides of the walls, instead of squarely against them. This natural position is the redeeming feature of the cervical border, and has made it possible for many botch operators to make fillings that were successful at this point. I am under the impression that some operators, appreciating this fact of the square application of the instrument along this border, use thicker mats or masses of gold than they are able to condense thoroughly, even though considerable mallet force is applied. I have repeatedly seen fillings that were solid and fine in other parts, and yet along this border were soft and even imperfect. In fact, I think we are all inclined to work too rapidly in packing the gold here, and also that we use the gold in too large masses.

For this purpose I have devised and used for many years plugger points which are adapted to bring pressure on the edge of the cavity, and which, being made flat on the side which rests against the matrix, insures a very perfect adaptation of the gold. These are made for the cavities on the anterior and posterior sides of the teeth respectively. It is not easy to describe their exact shapes, but I think they will be appreciated when seen.

There has been much said for and against a loose-fitting matrix, —that is, one that stands off from the tooth somewhat so that the gold can be packed a little beyond the cavity, thereby insuring a better adaptation at the edge. There is some reason in this, and yet it may be an open question if the time spent in finishing such a filling were given to packing the gold in smaller pieces, and with the instrument just described against a close-fitting matrix, an equally good edge would be secured.

The peculiar "rake" of these points makes it possible to make a perfect edge if one will be patient and careful. Of course, it is assumed that the matrix is bulged to conform to the natural shape of the tooth. If an equally good edge can be made with a close-fitting matrix, and as quickly, when the whole operation is considered, there is a gain in saving the patient the disagreeable task of trimming the gold, which generally results in disturbance of the gum from the use of files or chisels and the dreaded sand-paper strips. In the early years of the matrix I was shy of it, fearing the making of bad margins, but since having the plugging instruments described, my use of the matrix has increased, while my dependence on the separator has lessened.

A word more now, even at the risk of repetition, in reference to the cervical border. If gentleness and close care in filling are to characterize our method, and some soft or semi-soft gold is to be our main reliance, then I think we need not cut so radically in the preparation of this border.

Of course, this will be for the purpose of avoiding the encroachment upon the thin enamel, and to save the disturbance of the gum, as well as to make the operation easier for both patient and operator, as before stated. If it is said that we should not stop short of a radical operation, no matter what effort to the operator or discomfort to the patient, if it only betters an operation, then I reply, "Is it a better operation?" And when all the conditions are considered, I answer, "I think not." Nature is a safe guide, and I think we work well when we strive to leave a natural condition of the gum and of the teeth at the cervical margin.

It is perhaps a little curious that after the lapse of a quarter of a century I should have to ask for moderation in the application of a method which then seemed so necessary. But the confidence of youth is beyond belief. It is a divine quality, and springs from something in the blood that scorns delay and will not brook interference with the attainment of that perfection which is its dearest dream. What would we give to know again its divine thrill! To be carried by it beyond the bounds of reason, to know again its superb audacity. To scorn the wisdom of the universe in the confidence of its own dictum. But true to the inherent force that guides its movement, the pendulum ever swings back, and human progress is attained by rhythmic movements, that like the rising tide recedes only to creep a little higher on its return.

So I will not complain of radicalism, though I feel that now I know its danger. I now come naturally to the consideration of plastics in their relations to this border. And here let me tell you frankly that, for my appearance before you, I selected this subject partly for one particular purpose,—namely, to emphasize the danger of using oxyphosphate of zinc at this border without preceding it by some insoluble and indestructible substance. Oxyphosphate of zinc has been one of the most valuable acquisitions in dentistry, and it has been one of the most dangerous. I shall feel well repaid for whatever effort it has cost me, if I can only succeed in making a few members of the profession appreciate a little more fully the danger of using oxyphosphate of zinc along the cervical border. It may be that it is not generally used in this place, and that my anxiety is unnecessary, but it is my observation that it is generally so used here by most operators in this country and by those who practise abroad, and without being preceded by any other indestructible substance. I am sure there is not one of us who would not to-day be crippled in our practice by the withdrawal of oxyphosphate of zinc, and at the same time I believe there are not many of us who fully appreciate its danger.

In the beginning I said that the cervical border was the most inaccessible and most vulnerable of any cavity border in the mouth. If against this border you will use a substance so liable to chemical disintegration as oxyphosphate of zinc, you will have intensified the conditions of danger, and although you have delayed the evil hour of the tooth's destruction, you have established a set of conditions that in time will bring it about with almost scientific certainty.

It would not be easy to estimate the number of pulps that have become exposed by the treacherous washing out of this material at the cervical border, thus allowing decay to go on quietly, without giving any intimation of its existence until too late. And the same may be said of the copper amalgam which was so much used a few years ago, and which dissolved at the cervical border in nearly the same manner. It left a dirty trail, and the memory of it is a nightmare. But we are not concerned with it in this paper, and let us hope it is a thing of the past.

What material shall we use, then, at the cervical border, if we have decided to put an oxyphosphate filling on an approximate surface? It depends upon the teeth. If in the front teeth, it should be gutta-percha or gold. If in the bicuspids or molars, it should be gutta-percha, amalgam, or gold. I do not include tin, as that will be mentioned later.

The need of any indestructible substance is not so great in the front teeth as in the bicuspids and molars. Any one of considerable experience will understand this. The teeth are not so broad and the cervical border not so sheltered, and the conditions taken altogether are not so treacherous. Gutta-percha is quickly applied to all these cervical borders, and has the great advantage of being safe. Though, of course, in a few years after expanding and bulging out so that it must be trimmed off, it will finally rot and have to be renewed. It is a very safe substance to use, however, as any bulging and rotting will be detected, owing to the fact that the oxyphosphate placed on it will wear away and have to be renewed in two or three years at the latest, and at each renewal the exact condition of the cervical border can easily be ascertained.

This combination for very badly decayed teeth, particularly bicuspids, becomes in my judgment a very wise one to adopt. There will be generally two or three renewals of the oxyphosphate to one of gutta-percha. Teeth treated in this way may not increase one's reputation, because when the oxyphosphate wears away patients will come in saying the filling is out, but the operator can hear this undisturbed, because he knows at the cervical border it is safe and sound.

Of all the substances that can be used at this border in badly decayed teeth that must be treated with plastics I believe gutta-percha the safest. I will not say that I think it in all respects the nicest, for the bulging or the rotting will be noticed by careful patients who have the habit of passing the silk; but for absolute safety I think it is unsurpassed by any other substance, and for the reason that with it such a perfect adaptation can be made to the walls of the tooth, and because it has the preservative quality.

For many years I have in many cases protected the gutta-percha from wear by covering it with amalgam instead of oxyphosphate of zinc. In such cases I have filled the cavities nearly full of gutta-percha and then covered them on the grinding surface only with a rather thin filling of amalgam. I have known such fillings to last many years, but there is one danger,—namely, that of the expansion of the gutta-percha causing the thin walls of the tooth to split off. This danger is not so great, however, if the amalgam is only used on the grinding surface and the gutta-percha is left free on the whole approximal surface.

Gold can be used along this border to great advantage in many teeth so badly decayed that it could not wisely be used for the complete filling. The de Trey and similar forms of gold are very well suited for this purpose, as they can be so easily and accurately applied, as before stated. Amalgam for this purpose in many cases becomes invaluable. I have used it with a constantly growing confidence in its value.

Of course, you will understand that I am speaking of these three substances—gutta-percha, gold, and amalgam—only as foundations at the cervical border for oxyphosphate of zinc. This is no place for the discussion of the relative value of filling-materials, only as applied to the cervical border. I have not included tin, for while in some places I consider it the most perfect of all filling-materials, I do not trust it in sheltered places on proximate surfaces, and for the reason, of course, that it undergoes chemical dissolution. For this reason, many years ago, when it was advocated as well suited, pure or rolled with an alternate leaf of gold, for use along the cervical border, I distrusted it and used it but little. The few teeth that I ever filled in that way were afterwards repaired by replacing the softened tin by either gold or amalgam. If tin can be packed near to the edge of the enamel, and gold placed over it so that no tin is left exposed when the filling is finished, a durable filling may be expected. This is now done by Dr. Shumway, who finds that gold adheres to tin when rubbed onto it with ivory points. He rolls the tin in a compact roll and, cutting it in short pieces, anneals it over mica nearly to its melting-point, and after packing in the cavity he burnishes the surface with an ivory point and then rubs cohesive gold onto it. He claims the union between the metals is sufficient to make a fine filling, the tin being protected from wear or disintegration by the veneering of gold. To be successful, he says, the tin must be annealed and the filling protected by the rubber dam from even the breath. The results of a clinic given last Tuesday before the New York Odontological Society showed that he makes no idle claim.

This is a proper place to add a few words in reference to the repair of gold fillings that fail at the cervical border. Purposely I did not allude to it while speaking of cavities that were to be filled with gold throughout. I have no means of knowing what is the general practice in these cases,—whether it is that of removing the whole gold so as to get access to the defective place, or that of simply cutting out the decay and repairing with gold or amalgam, or even with gutta-percha. My own practice has been almost invariably that of repairing the defective place, if the body of the filling is good. If the gum in such cases cannot be held away by the rubber dam, it can be by a suitably shaped instrument, so that access can be had to the cavity from both the buccal and lingual sides, and the operation becomes a very simple one either for the use of gold or amalgam.

If a large gold filling requires repairing at this point, it is more satisfactory to do it with gold if it can be done accurately; but if the defective place is inaccessible, a thoroughly durable operation can be made with amalgam. I consider this one of the best uses to which amalgam can be put. I have done so many with it in this way, and have seen them last so many years, that I have come to have great confidence in it for these places. The result is somewhat like that attained by using amalgam in the upper half or two-thirds of the cavity, and finishing with gold at the same sitting,—a method described first, I think, by Dr. Dwight M. Clapp, of Boston, and one that in my judgment has great merit in some cases.

The repairs I have in mind are only slight, however, and do not let the amalgam come in sight. The ability to make these repairs so easily is one reason why I am not so ready now as formerly to cut the teeth away so that each cavity on the proximate surface shall go under the gum.

There may be objection on the part of some to the use of amalgam for these repairs. I think the inherent objections to amalgam do not apply with the same force in these cases as when used for large operations.

Gold is very clean and beautiful, and amalgam is dirty and repugnant, but a man's professional duty to his patient should lift him above such considerations, and he should have the courage of his convictions, and the independence to use the means that will most easily and most surely make his work a success.

I have unstinted admiration for those who reject amalgam, in particular, because it is a bad material in general; and yet there is a higher ground still to take, and it is reached when, laying aside all prejudice, one has the courage to use it in those few cases where for practical use and service it is the best thing we have yet found.

Sufficient unto the day is the evil thereof, and in this connection I am tempted to go out of my way to allude to a practice, much talked of a few years ago of anticipation of decay, by cutting between the approximal surfaces of good teeth. It had a grain of truth, but where is it to-day? Fortunately relegated with all the other fads of the enthusiasts to the limbo of the past. Besides disturbing the gum, it transferred the point of contact to to the cervical border, after the teeth had readjusted themselves, and made the conditions still worse when decay occurred.

I have now a word to add in reference to the illumination of the cervical border. On dark days I concentrate the light by the condensing lens, held by an adjustable rod attached to an upright, screwed into the iron frame of the movable bracket attached to the S. S. White Wilkerson chair. This intensified light, when it is possible, is reflected into the cavity by the ordinary mouth-mirror held in the left hand, or held by the attendant. Sometimes I reflect this concentrated light, or ordinary daylight, by a little mouth-mirror held by a rod attached to a weighted standard, which sets on the tray. The mirror is attached by a ball-and-socket joint, so that it can be set at any angle. For one who has no attendant this is convenient, as it leaves the left hand free to hold the gold in place in commencing a filling. These devices are somewhat in the way, but if one can become accustomed to them they are a real help. I have also used the electric light, but I have thought it rather trying to the eyes.

In closing, I wish to call attention to a certain form of matrix which I have found indispensable in the use of plastics at the cervical border. It is one which I call a band-matrix, as it is attached to a handle which is to be held in the left hand. The matrices are made of thin steel with a lug soldered on the end which is to be placed on the lingual side of the teeth, and the handle soldered on the other. Some of them I have made from a single piece of steel, as the soft solder is affected by the mercury in amalgam filling, and they sometimes become unsoldered. If hard solder is used, so much heat is required that the temper of the steel is lost, and they do not keep their shapes as well as when they retain their spring temper. Those made from a single piece of steel are very durable. It is not, however, easy to make them wide enough for some cases, therefore I have some of them made by soldering both with hard and soft solder. I have tried various metals, but I find that steel having a spring temper is best. The only objection is that in using oxyphosphate of zinc there will be some corrosion of the steel, but this can be partly overcome by touching the surface of the matrix on a sponge charged with oil before using. These matrices have one advantage over all others I have ever seen. Being held by the left hand, the operator has perfect control of them. The lug resting against the adjoining tooth near the gum secures a close fit at the cervical wall of the tooth to be filled, and by means of the handle the upper side of the matrix can be turned out and away from the tooth in such a manner as to give access to the cavity. After the filling is partly in, the matrix can be turned up to the tooth so that the contour can be kept. This adaptability of the matrix through the medium of the left hand makes it, in my practice at least, indispensable. The manner in which it opens up the cavity to light, as well as for the reception of the filling, must be seen to be appreciated. By pulling on it the lug draws it closely down at the cervical wall and holds it there firmly, and at the same time it can be turned off at the upper edge so as to reach the adjoining tooth, even though it is some distance away.

For this reason it is invaluable in cases where it is desired to exaggerate the contour of a tooth in order to close up a space. It is applied in an instant, and can be taken out and put back at any time, even though the filling is only partly in. For plastics it is almost perfection, and for gold it is also of great value. I have become so used to it that I use it for many of my gold fillings, in fact, for nearly all, where I can spare the left hand to hold it. It is so adaptable to the will of the operator that, having once become used to it, I cannot understand how it could be given up. I presented this matrix, with the illuminating devices described above, before the New York Odontological Society in 1893, and they were illustrated in the INTERNATIONAL DENTAL JOURNAL of that year. They have never been manufactured for the profession, and are therefore but little known.

I have to ask your indulgence for having treated this subject of cervical borders as if you were students, but I saw at once that it is one of infinite technical detail, and there seemed to be no other way. If I have so often described my own method, it has been because I could not in any other way so easily state what I believe to be the best practice. Emerson said, "Tell me what you do, and I will understand all the rest."

